# Human Pluripotent Stem Cells to Model Islet Defects in Diabetes

**DOI:** 10.3389/fendo.2021.642152

**Published:** 2021-03-22

**Authors:** Diego Balboa, Diepiriye G. Iworima, Timothy J. Kieffer

**Affiliations:** ^1^ Regulatory Genomics and Diabetes, Centre for Genomic Regulation, The Barcelona Institute of Science and Technology, Barcelona, Spain; ^2^ Department of Cellular and Physiological Sciences, University of British Columbia, Vancouver, BC, Canada; ^3^ School of Biomedical Engineering, The University of British Columbia, Vancouver, BC, Canada; ^4^ Department of Surgery, University of British Columbia, Vancouver, BC, Canada

**Keywords:** diabetes, insulin, modeling, stem cells, genetic defects, insulin secretion

## Abstract

Diabetes mellitus is characterized by elevated levels of blood glucose and is ultimately caused by insufficient insulin production from pancreatic beta cells. Different research models have been utilized to unravel the molecular mechanisms leading to the onset of diabetes. The generation of pancreatic endocrine cells from human pluripotent stem cells constitutes an approach to study genetic defects leading to impaired beta cell development and function. Here, we review the recent progress in generating and characterizing functional stem cell-derived beta cells. We summarize the diabetes disease modeling possibilities that stem cells offer and the challenges that lie ahead to further improve these models.

## Introduction

More than 450 million people worldwide are diagnosed with diabetes, a number unfortunately expected to increase dramatically in the next decades (1). Diabetes unfolds when the pancreatic beta cells fail to secrete enough insulin to meet physiological demand, resulting in abnormally high blood glucose levels. Our understanding of the distinct molecular mechanisms that lead to beta cell failure in the different types of diabetes has remarkably improved thanks to progress in the genetic characterization of people with diabetes and the development of animal and cellular models (2). Among these models, the generation of islet cells from human pluripotent stem cells is gaining traction as a useful approach to dissect diabetes molecular mechanisms (3). In this review, we aim to summarize recent progress in diabetes disease modeling using human pluripotent stem cells, discussing current limitations and potential improvements. We particularly focus on advances in functional islet cell generation, and how these cells may be utilized to study beta cell insulin secretory defects.

Beta cell failure leading to diabetes occurs in different ways. While in type 1 diabetes, beta cells are destroyed by cytotoxic T lymphocytes (4), in type 2 diabetes, which represents 90% of all diabetes cases, the beta cells are dysfunctional as a result of maladaptation to elevated demand for insulin secretion, usually in the context of systemic insulin resistance (5, 6). Both type 1 and type 2 diabetes (T2D) result from interactions between a polygenic background and environmental factors like viral infections or obesity (7). Other forms of diabetes that are less frequent result from highly penetrant monogenic mutations that impair beta cell development and/or function. They can manifest at birth, transiently or permanently, in what is known as neonatal diabetes, or in the young adult (10–25 years of age), termed maturity onset diabetes of the young (MODY) (2, 8). While genetic variants in ~30 loci are associated with neonatal diabetes and MODY, over 50% of clinically diagnosed cases remain genetically unexplained despite continuous efforts to find causative genetic variants by using genome sequencing (9, 10).

The characterization of the genetic defects associated with these different types of diabetes has improved the understanding of the molecular mechanisms that trigger or increase the risk for this disease. Genome-wide association studies have so far identified over 400 association signals across ~200 loci associated with T2D. These genetic variants are particularly enriched in coding and non-coding genomic regions characteristic of pancreatic islet cells, highlighting their central role in the development of diabetes (11–13). Interestingly, several genetic variants associated with T2D are in loci of genes that are also mutated in cases of neonatal diabetes and MODY [e.g. *KCNJ11* (14, 15), *HNF1A* (16, 17), *GCK* (18, 19)]. These genes are critical for beta cell function and the severity of the disease is determined by the precise molecular mechanism disrupted by the particular genetic variant and its functional impact. There is a spectrum within diabetes in which the pathogenetic mechanisms might range from protein-truncating mutations causing neonatal diabetes due to pancreas developmental failure (20, 21), to increased T2D risk due to regulatory variants modulating adult islet cell function (22, 23). While genetic studies have identified numerous candidate genetic variants associated with different types of diabetes, functional validation of their impact on glucose homeostasis requires models that recapitulate as faithfully as possible human islet physiology.

Rodent animal models have provided abundant knowledge of pancreatic development and beta cell physiology. The generation of genetically modified mouse models have contributed to understanding the role of genes involved in these processes (21, 24). However, animal models have inherent limitations due to key differences with humans at the genetic and physiological level (25, 26). Primary human islets obtained from the pancreas of cadaveric donors are a valuable research material to study diabetes. They have been used to study particular aspects of human islet physiology (27) and to understand how genetic variation affects islet function (28). However, human islet preparations are scarce and exhibit considerable variability in terms of purity, function, and cell type composition after isolation (29–32). Furthermore, isolated human islets are challenging to keep in culture for extended periods of time, and the ability to use them to study the effect of particular genetic variants is limited by the current capabilities to genetically manipulate them. As an alternative, there have been many attempts to generate immortalized human beta cells resulting in the derivation of several cell lines that are now widely used in research. They constitute a renewable source of beta-like cells that can be used to perform diverse *in vitro* experiments. In particular, EndoC-βH lines have proven to be a particularly useful model since they present glucose-stimulated insulin secretion *in vitro* and are transcriptomically similar to primary beta cells (33, 34). Such lines can be utilized to study the impact of particular genetic variants and perform drug screenings since they are amenable to genetic modification and other perturbations (23, 34). A drawback of these cells is that they are aneuploid, which can be a confounding factor for genetic studies (35). They also proliferate, which compromises the functional characteristics of adult beta cells (36, 37). This has been resolved in conditionally immortalized versions of this cell line where the SV40LT oncogene used to transform them can be removed by inducible genetic recombination (37, 38); these cells continue to be a useful resource for the field.

Differentiated human pluripotent stem cells (hPSCs) represent another source of human beta cells. hPSCs can be derived from human embryos (human embryonic stem cells, hESCs) (39) or from somatic cells *via* nuclear reprogramming (human induced pluripotent stem cells, hiPSCs) (40). Notably, hiPSCs can be obtained from somatic cells of people that carry diabetes-associated genetic variants. By doing so, pluripotent cell lines preserving the donor genetic background can then be differentiated *in vitro* into particular cell types to model the molecular consequences of the genetic variant under study (41). Importantly, hPSCs are amenable to different genome editing approaches, facilitating the correction or introduction of desired genetic variants. This is a useful approach to generate optimal isogenic controls or to create new models when donor sources are not available (42).

Here we discuss the possibilities of using hPSCs to model the impact of diabetes-associated genetic variants on the physiology of the beta cell, focusing on the molecular mechanisms impairing insulin secretion.

## Beta Cell Insulin Secretion Defects

All forms of diabetes have in common the ultimate dysfunction of the pancreatic beta cells and the consequent inadequate circulating insulin levels. Beta cells constitute about 60% of the cells in the human islets. They are highly intermingled with the other endocrine cells, in particular with glucagon producing alpha cells, the second most abundant type, a configuration that is crucial for the optimal function of the beta cells (43, 44), and somatostatin secreting delta cells that dampen the release of both insulin and glucagon (45). The particular organization of human islet cells is remarkably heterogeneous, with variable islet size and cell type composition across parts of the pancreas, but also showing important variation across individuals and from birth to adulthood (29–31).

**Figure 1:**
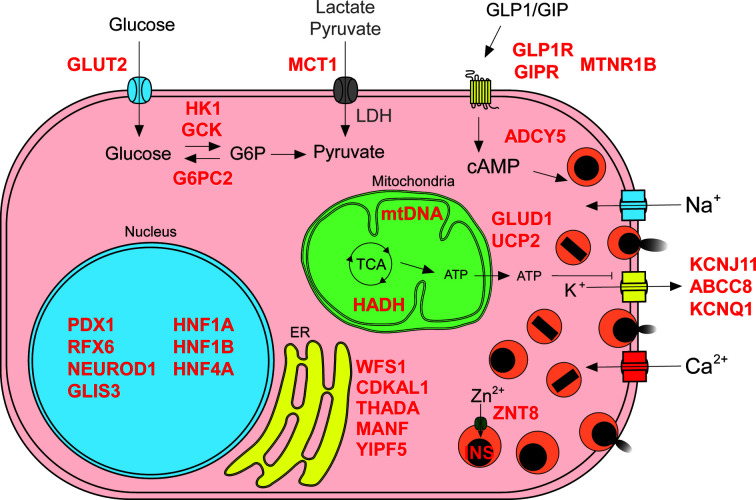
Insulin secretion molecular mechanisms affected in diabetes. Genetic defects can impair different processes involved in regulated insulin secretion (known genes affected in red text): glucose import and metabolism (G6P, glucose 6-phosphate; LDH, lactate dehydrogenase; TCA, tricarboxylic acid cycle; abnormal beta cell metabolism of non-glucose carbon sources due to failure in silencing of disallowed genes depicted in gray text), membrane depolarization, membrane receptors, insulin synthesis and secretion, endoplasmic reticulum (ER) homeostasis, and transcriptional regulation.

In conjunction with glucagon secreting alpha cells, beta cells keep human fasting blood glucose concentrations around 5 mM, normoglycemia, by adjusting their insulin secretion output (44). Beta cells are fine-tuned glucose sensors with an intricate machinery that enables them to respond with exquisite precision to deviations from normoglycemia, such as during meals, to minimize glucose excursions (46, 47). Genetic variants that result in the disruption of these molecular mechanisms impact the capacity of beta cells to secrete insulin in a regulated manner. These can cause reduced insulin secretion, leading to the development of different forms of diabetes, or increased insulin secretion (hyperinsulinism) (20, 48). We discuss some of these in detail below (summarized in **Table 1** and **Figure 1**).

**Table 1:** Genetic defects leading to dysregulated beta cell insulin secretion.

Mechanism affected	Genes	Impact of genetic defect	Type of disease	References
**Glucose import and metabolism**	*GCK*	Reduced or increased glucokinase activity results in abnormal glycolytic flux, ATP generation, and insulin secretion	ND, MODY, T2D, CHI	(18, 19, 49)
	*G6PC2*	Loss of function mutations are associated with reduced fasting glycemia	T2D	(50)
	*SLC2A2 (GLUT2)*	Loss of function mutations result in impaired glucose uptake	ND, T2D	(11, 51)
	*HK1*	Abnormal silencing of HK1 in beta-cells results in increased glycolytic flux, ATP generation and insulin secretion	CHI	(52)
	*SLC16A1 (MCT1)*	Promoter mutations impair SLC16A1 silencing in beta-cells, resulting in abnormal pyruvate uptake, increased ATP generation, and insulin secretion	CHI	(53)
	*GLUD1*	Gain of function mutations result in increased entrance of glutamate in TCA cycle, increased ATP generation, and insulin secretion	CHI	(54)
	*HADH*	Loss of function mutations result in abnormal activation of GLUD1, increased glutamate into TCA, ATP generation, and insulin secretion	CHI	(55, 56)
	*UCP2*	Gain or loss of function mutations alter the mitochondrial uncoupling activity of UCP2, resulting in abnormal ATP generation and insulin secretion	T2D, CHI	(57, 58)
	mtDNA	Mitochondrial DNA mutations impair oxidative phosphorylation, ATP generation, and insulin secretion	–	(59)
**Membrane depolarization**	*KCNJ11*	Gain or loss of function mutations result in abnormal closure or opening of the channel, altered membrane depolarization, and insulin secretion	ND, MODY, T2D, CHI	(55, 60–62)
	*ABCC8*	Gain or loss of function mutations result in abnormal closure or opening of the channel, altered membrane depolarization, and insulin secretion	ND, MODY, T2D, CHI	(14, 15, 55, 63, 64)
	*KCNQ1*	Genetic variants in this locus are associated with T2D risk.	T2D	(11, 65)
**Membrane receptors**	*GLP1R*	Genetic variants in this locus are associated with lower fasting glucose levels. Altered GLP-1 signaling affects amplification of insulin secretion.	T2D	(66)
	*GIPR*	Genetic variants associated with reduced GIP signaling, impair incretin-mediated amplification of insulin secretion.	T2D	(67)
	*MTNR1B*	A genetic variant increasing melatonin signaling lowers cAMP levels, inhibiting insulin secretion.	T2D	(68)
**Insulin synthesis and secretion**	*INS*	Loss of function mutations disrupt INS protein synthesis, folding, transport or bioactivity.	ND, MODY, T2D	(11, 69)
	*SLC30A8 (ZNT8)*	Different coding genetic variants increase risk or protect against T2D.	T2D	(70–72)
	*ADCY5*	Non-coding genetic variant reduces ADCY5 expression, which couples glucose to cAMP generation, increasing T2D risk.	T2D	(73, 74)
**ER homeostasis**	*WFS1*	Loss of function mutations lead to elevated ER stress and beta cell dysfunction.	ND, T2D	(75)
	*CDKAL1*	Loss of function mutations induce beta cell ER stress and hypersensitivity to glucotoxicity and lipotoxicity.	T2D	(76)
	*THADA*	Coding genetic variants associated with increased T2D risk.	T2D	(77)
	*MANF*	Loss of function mutations cause childhood diabetes and a neurodevelopmental disorder.	ND, T2D	(78, 79)
	*YIPF5*	Loss of function mutations impaired ER-to-Golgi trafficking leading to increased beta cell ER-stress.	ND	(80)
**Transcriptional regulation**	*PDX1*	Loss of function mutations impair transcriptional regulation of pancreatic development and adult islet cell function.	ND, MODY, T2D	(81–84)
	*RFX6*	Loss of function mutations impair transcriptional regulation of pancreatic development and adult islet cell function.	ND, MODY	(85–87)
	*NEUROD1*	Loss of function mutations impair transcriptional regulation of pancreatic development and adult islet cell function.	ND, MODY	(88–90)
	*GLIS3*	Coding and non-coding genetic variants impair transcriptional pancreatic development and adult islet cell function	ND, MODY, T2D	(91–93)
	*HNF1A*	Coding and non-coding genetic variants impair transcriptional pancreatic development and adult islet cell function.	MODY, T2D, CHI	(16, 17, 94)
	*HNF1B*	Coding and non-coding genetic variants impair transcriptional pancreatic development and adult islet cell function.	ND, MODY, T2D	(95, 96)
	*HNF4A*	Coding and non-coding genetic variants impair transcriptional pancreatic development and adult islet cell function.	MODY, T2D, CHI	(54, 97)
	*TCF7L2*	Coding and non-coding genetic variants impair transcriptional pancreatic development and adult islet cell function.	T2D	(98, 99)

ND, Neonatal Diabetes; MODY, Maturity Onset Diabetes of the Young; T2D, Type 2 Diabetes; CHI, Congenital Hyperinsulinism.

Summary of genetic variants that impact on molecular mechanisms involved in insulin secretion by the beta cell, classified by the mechanism affected and detailing the impact of the genetic defect.

Islet cells are profusely vascularized, and this facilitates the sensing of circulating blood glucose levels. Glucose is imported into human beta cells primarily *via* glucose transporters 1 (GLUT1) and 3 (GLUT3). Glucose transporter 2 (GLUT2), the main transporter in rodent beta cells, is expressed at lower levels in human beta cells (100). Interestingly, genetic variants associated with T2D are found in *GLUT2* (51) suggesting an important role in human beta cells.

Imported glucose is phosphorylated by glucokinase (GCK), a low affinity hexokinase. Diverse genetic defects in *GCK* lead to different kinds of insulin secretion phenotypes, resulting in a range of disease severity, from neonatal diabetes and MODY, to increased T2D risk and congenital hyperinsulinism (18, 19, 49). Other regulators of glucose phosphorylation have also been implicated in insulin secretion dysregulation. For example, glucose-6-phosphatase 2 (*G6PC2*) harbors genetic variants associated with reduced fasting glycemia (50), while the abnormal beta cell expression of “disallowed gene” (101) hexokinase I (*HK1*) has been linked to congenital hyperinsulinism (52).

Once phosphorylated, glucose is retained within the beta cell and it enters the glycolytic pathway to generate pyruvate. Pyruvate is then further oxidized in the mitochondrial tricarboxylic acid cycle, generating abundant chemical energy in the form of ATP, and thus increasing the ATP to ADP ratio. This results in depolarization of the beta cell membrane *via* closure of the ATP-sensitive potassium channels (K^+^_ATP_), triggering insulin secretion. Thus, oxidative metabolism of pyruvate constitutes a crucial coupling process enabling regulated insulin secretion (102). As an alternative to this canonical, one-state model of insulin secretion, recent work by Lewandowski et al. proposes a dynamic model in which beta cells in high glucose conditions oscillate between two states: a biosynthetic state in which conversion of ADP and phosphoenolpyruvate into ATP and pyruvate by pyruvate kinase results in closure of the K^+^_ATP_ channels, triggering exocytosis, followed by a state of active oxidative phosphorylation that supports the elevated ATP to ADP ratio sustaining membrane depolarization until exocytosis-associated processes reduce the ATP levels (103, 104). Activators of pyruvate kinase resulted in potentiated GSIS in both rodent and human islets, suggesting that pyruvate kinase may be a potential therapeutic target for T2D (53). Different defects related to the abnormal incorporation of metabolites into the tricarboxylic acid cycle are associated with congenital hyperinsulinism and T2D. Impaired silencing of the pyruvate and lactate transporter *SLC16A1* (*MCT1*), a beta cell disallowed gene, results in congenital hyperinsulinism (53). Gain of function missense mutations in *GLUD1*, glutamate dehydrogenase, or loss of function mutations in hydroxyacyl-coenzyme A dehydrogenase (*HADH*) increase incorporation of glutamate into the TCA cycle leading to congenital hyperinsulinism (54–56). Also, genetic variation in the mitochondrial uncoupler *UCP2* has been associated with T2D and congenital hyperinsulinism (57, 58). In addition, mitochondrial DNA mutations that impair ATP generation cause syndromes that present with diabetes of variable severity (59).

The increase of ATP to ADP ratio triggers the closure of membrane K^+^_ATP_ channels, formed by the proteins KCNJ11 and ABCC8 (105). Channel closure leads to depolarization of cell membrane and opening of additional Na^+^ and Ca^2+^ channels. Ca^2+^ influx crucially couples membrane depolarization with insulin exocytosis, in a process mediated by the Ca^2+^-sensing proteins synaptotagmins which trigger the fusion of insulin granules with the plasma membrane (105). Gain of function mutations in *KCNJ11* and *ABCC8* resulting in constant channel opening are the most common cause of neonatal diabetes due to islet physiology defects (15, 60, 63). Genetic variation in these genes can also cause MODY and increased T2D risk (61, 62). Loss of function mutations that result in constant K_ATP_ closure, or impair its trafficking to the membrane, lead to congenital hyperinsulinism (14, 55). Furthermore, genetic variants in the voltage-gated K^+^ channel KCNQ1 are associated with increased T2D risk (65).

Influx of Ca^2+^ ions into depolarized beta cells induces insulin exocytosis by activating the synaptotagmins and SNARE proteins that regulate the fusion of insulin granules with the plasma membrane (106). This exocytosis machinery is not only regulated by the intrinsic pathway triggered by membrane depolarization but is also critically modulated by the intracellular levels of cyclic AMP (cAMP), in what is known as the amplifying pathway (107). Incretin hormones glucagon-like peptide-1 (GLP-1) and glucose dependent insulinotropic polypeptide (GIP), released by intestinal enteroendocrine cells, potentiate insulin secretion upon binding to their cognate G-protein coupled receptors in the membrane of beta cells (108, 109). This binding results in the generation of cAMP and activation of protein kinase A pathway resulting in augmented K^+^ channel inhibition, Ca^2+^ influx, and insulin exocytosis (110). GLP-1 also regulates the alpha cells in a glucose-dependent manner, inhibiting glucagon at high glucose levels, and thereby further contributing to glucose homeostasis (111, 112). GIP stimulates glucagon secretion in a glucose-dependent manner in healthy individuals, with enhanced activity at lower glycemia (113). However, GIP stimulates glucagon secretion even in the presence of hyperglycemia in subjects with T2D, and thereby could contribute to the pathogenesis of T2D (113). Genetic variants found in both the incretin receptors genes *GLP1R* and *GIPR* have been associated with increased and decreased risk of T2D (66, 67). Melatonin receptor 1 B (*MTNR1B)*, another G-protein coupled receptor present in the membrane of beta cells, has also been linked to T2D. A genetic variant increasing the expression of *MTNR1B* has been shown to lower cAMP levels in beta cells, leading to reduced insulin secretion (68). Furthermore, a genetic variant that results in reduced expression of the adenyl cyclase five (*ADCY5*), which regulates beta cell cAMP levels, has been associated with increased risk of T2D (73, 74).

Insulin protein is synthesized in remarkable amounts, representing up to 50% of beta cell total protein synthesis (114), and imposes a high demand on the protein folding machinery of the endoplasmic reticulum (ER). These processes are controlled by the unfolded protein response (UPR) pathway, which is highly efficient in beta cells in order to cope with the insulin biosynthesis-induced ER-stress (115, 116). After potassium channel defects, coding mutations in the insulin gene are the second most common cause of neonatal diabetes due to beta cell dysfunction. These missense mutations cause defects in proinsulin translation, folding, or processing, and may induce high levels of ER-stress that leads to dysfunction of the beta cell. Some *INS* coding mutations can also cause MODY (69) and genetic variants in the *INS/IGF2* locus have been associated with T2D increased risk (11). The fine-tuning of ER-stress levels in beta cells is crucial for the proper functioning of these busy insulin factories. Coding mutations in components of the UPR pathway can cause neonatal diabetes or increased risk for T2D (e.g. *WFS1*, *CDKAL*, *THADA*, *MANF*, *YIPF5*) (11, 75–80). Processed proinsulin molecules are tightly packaged as Zn^2+^ complexed crystals in dense core exocytotic granules. Genetic variants in the Zn^2+^ transporter *SLC30A8* (*ZNT8*), present in the membrane of insulin granules, have been associated with T2D susceptibility (70, 71). A rare loss of function mutation in ZNT8 protects against T2D, making this Zn^2+^ transporter a potentially interesting therapeutic target (72).

While coding and non-coding genetic variants linked to diabetes often impact mechanisms regulating insulin secretion from beta cells, some of them perturb the development of the pancreas, islets, and beta cells themselves (23, 48). The expression levels of insulin secretion machinery components is controlled by transcription factors that conform gene regulatory networks governing the beta cell transcriptional program (117). However, many of these transcription factors are also involved in regulating beta cell development (e.g. FOXA2, PDX1, MNX1, NEUROD1, PTF1A, HNF1A, RFX6) and genetic defects in their loci might lead to a wide variety of diabetes phenotypes (20). While highly damaging transcription factor mutations can cause developmental defects leading to pancreatic agenesis and neonatal diabetes, other genetic variants with milder effects might lead to MODY with different clinical features and penetrance (17, 81, 118), increased T2D risk (91, 98), or even congenital hyperinsulinism (54, 94). Epigenetic profiling of human islets has enabled the characterization of their regulatory landscape, showing that dense enhancer areas are enriched in genetic variants associated with T2D risk (13). Furthermore, a recent study characterizing human islet chromatin architecture resulted in the identification of 3D higher-order hubs of enhancers and promoters (23). These regions are enriched for genetic variants that impact on the heritability of islet-cell traits. We summarize in **Table 1** a list of genes that harbor genetic variants specifically linked to dysregulated insulin secretion. The impact of a given genetic variant will depend on how deleterious it is for a particular mechanism controlling insulin secretion, thus determining the diabetes phenotype and the possible therapeutic interventions. Given the wide spectrum in the functional consequences of coding and non-coding genetic variants, we need suitable research models that enable precise dissection of the detailed mechanisms by which these genetic variants impair human islet physiology.

## Modeling Insulin Secretion Defects Using Stem Cell Derived Islet Cells

**Figure 2:**
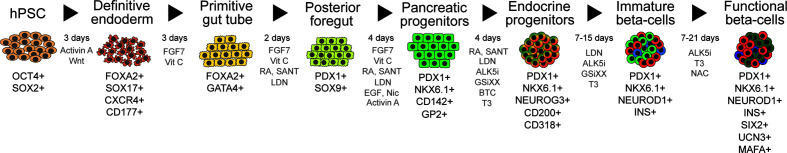
Multistage differentiation protocol to generate functional islet cells from human pluripotent stem cells. Current islet cell differentiation protocols mimic pancreatic developmental stages. Here we represent the commonly used stages [based on (121)], with their usual duration in days, together with cell markers used for the characterization of the differentiated cells (black text) and the cocktails of signaling molecules utilized to induce differentiation (gray text; FGF7, fibroblast growth factor 7; VitC, vitamin C, ascorbic acid; RA, retinoic acid; SANT, SANT-1, a sonic hedgehog signaling inhibitor; LDN, LDN-193189, a BMP inhibitor; EGF, epidermal growth factor; Nic, nicotinamide; ALK5i, a TGF-beta inhibitor; GSiXX, gamma secretase inhibitor used to inhibit Notch signaling; BTC, betacellulin; T3, triiodothyronine; NAC, N-Acetylcysteine).

The generation of hPSC-derived beta cells typically relies on differentiation protocols recapitulating the inductive signaling cues that instruct pancreatic development *in vivo*. These protocols have been devised based on knowledge gained from developmental biology, mostly using mouse models, that deciphered the dynamic signaling environment required for pancreas specification, endocrinogenesis, and beta cell formation (119, 120). With this information, different research teams have empirically determined the recipe of recombinant proteins and small molecules that reproduce developmental signals in a stepwise manner. These efforts have crystallized in differentiation protocols that make possible the efficient derivation of beta cells from hPSCs (**Figure 2**).

The first report demonstrating the feasibility of generating insulin-producing cells from human embryonic stem cells *in vitro* relied on an spontaneous differentiation approach (122). The first directed differentiation protocol was reported by D’Amour and colleagues from the company CyThera (now Viacyte Inc.). They devised a multistage, adherent culture differentiation protocol, that relied on a first step of efficient definitive endoderm induction (123), followed by four additional steps to induce primitive gut tube, posterior foregut, pancreatic progenitors, and hormone expressing cells (124). While this pioneer protocol generated relatively few insulin producing cells (about 7%), these cells became functionally mature after implantation into mice. Furthermore, the implanted cells were able to protect against streptozotocin induced diabetes (125).

These results sparked intense effort to develop improved protocols leading to more efficient and robust ways to obtain hPSC-derived beta cells over the next decade. Modulation of additional signaling pathways (e.g. FGF, TGF-beta, EGF, PKC) in a time-wise manner enhanced the differentiation efficiency of pancreatic progenitors and endocrine cells (126–132). However, detailed characterization of the hPSC-derived beta cells showed that these insulin expressing cells were frequently co-expressing other hormones, like glucagon or somatostatin, (usually termed as “polyhormonal” cells) (133–135). Polyhormonal cells had impaired glucose stimulated insulin secretion (134), aberrant epigenetic profiles (136), inappropriate glucose transporter GLUT1 expression, imbalanced K+_ATP_ channel subunit expression (137), and resembled human fetal beta cells at the transcriptomic level (138).

A critical realization was the importance of beta cell programming transcription NKX6-1 for beta cell development and functionality (139). NKX6-1 expression at the pancreatic progenitor stage of the differentiation was shown to be crucial for the generation of “monohormonal” beta cells, expressing insulin together with NKX6-1 (INS+NKX6-1+ beta cells) (140). Delaying NEUROG3 induction to later stages, when PDX1+NKX6-1+ progenitors are more abundant, increased the fraction of insulin^+^ glucagon^−^ beta cells (140, 141). Protocols generating functional beta cells *in vitro* from hPSCs were reported in 2014 (121, 142). Both differentiation protocols have similarities in the length, stages, and signaling cues used, resulting in abundant INS+NKX6-1+ beta cells. Endocrine cell differentiation was induced by a combination of ALK5 (a TGF-beta receptor) and NOTCH signal inhibitors. Thyroid hormone triiodothyronine (T3) was used to induce the expression of MAFA, a beta cell maturation marker (143, 144). The stem cell-derived beta cells secreted insulin in response to high glucose under static conditions, however, a more detailed analysis of dynamic insulin secretion and calcium influx showed the response was minimal compared to human islets (121). In both studies, the implantation of these functional hPSC-derived beta cells rescued diabetes in mice and had increasing levels of human insulin produced by the grafts over time.

These landmark reports demonstrated a viable path towards the generation of glucose-responsive hPSC-derived beta cells *in vitro*, despite the cells not matching the fine-tuned responses of human islets. It is important to recognize that human islets isolated from cadaveric donors, while presently used as the gold-standard control, have the limitation of considerable variability across islet preparations from different donors in terms of purity, cell-type composition, functionality, and expression of important beta cell markers (30, 121, 145).

Subsequent studies have built on these protocols and further refined them to achieve a higher percentage of hPSC-derived beta cells with better functional responses. For example, different studies have demonstrated how NKX6-1 expression can be increased by aggregating the pancreatic progenitors (146, 147) or by adding EGF and Nicotinamide (148). Also, Rho-associated kinase (ROCK) inhibitors were shown to boost the expression levels of NKX6-1 (149) and the numbers and maturation of hPSC-derived beta cells (150). ROCK inhibitor Y-27632 together with TGF-beta ligand Activin A was reported to induce the formation of endocrine cell enriched protrusions in a 3D-aggregate differentiation setup (151). Performing the differentiation in 3D suspension conditions, in an attempt to recapitulate the developing pancreas cytoarchitecture, has improved the generation of pancreatic progenitors and endocrine cells, as well as the reproducibility and scalability of the differentiation (141, 142, 146, 152).

Induction of endocrine cell formation in these differentiation protocols has relied on the modulation of NOTCH (using gamma secretase inhibitors), TGF-beta (ALK5 receptor inhibitors), and EGF (EGF and betacellulin ligands) signaling to trigger NEUROG3 expression. Interestingly, a newly developed 2D planar differentiation protocol generated cells with improved function using latrunculin A to depolymerize the cytoskeleton during endocrine induction, demonstrating that the cytoskeletal state of cells during differentiation can also modulate NEUROG3 expression. These cells were also able to reverse diabetes in STZ treated mice faster than cells generated with a 3D suspension protocol (153). Appropriate timing of NEUROG3 expression is important for beta cell lineage commitment. Its induction in pancreatic progenitor cells expressing PDX1+NKX6-1+ favors the generation of beta cells, while inducing at earlier stages will result in polyhormonal cells that appear to largely resolve into alpha cells (129, 140, 141, 148). Regulatory genomics analyses of embryonic and stem cell derived pancreatic progenitors identified TEAD and YAP as important regulators critical for pancreatic progenitors outgrowth (154). These effectors of the Hippo signaling pathway form part of the gene regulatory network that recruits pancreatic progenitor enhancers and controls their proliferation. Disruption of the TEAD-YAP complex with verteporfin results in reduced proliferation of mouse, zebrafish, and hPSC-derived pancreatic progenitors (154). Additional studies on the role of TEAD-YAP in pancreatic progenitors have shown that cell confinement prevents YAP nuclear accumulation and is a prerequisite for NEUROG3 upregulation (155). In this model, endocrinogenesis is triggered by the disruption of extracellular matrix signaling *via* integrin alpha 5, which maintains the expression of NEUROG3 repressor complex YAP1-TEAD4-HES1. Consistent with this, the use of verteporfin in stem cell-derived pancreatic progenitors resulted in reduced progenitor proliferation, increased NEUROG3 expression, and more C-peptide+ cells (155, 156).

Other approaches to improve the function of stem cell derived beta cells have relied on enrichment steps at various stages and controlling 3D cluster size. For example, enrichment of GP2+ pancreatic progenitors led to the generation of increased numbers of beta cells (157, 158), while enrichment of later differentiation stages using an INS-GFP reporter cell line or magnetic-based enrichment for ITGA1 improved the functionality of the stem-cell derived islet-like aggregates (159, 160). Optimal cluster diameter is important in order to avoid necrosis in the cell cluster core due to hypoxia, maintain a good surface to volume relationship, and is critical for glucose sensing and insulin release dynamics. Across mammalian species with different pancreas sizes, the diameter of islets averages 100–200 µm. The fact that mammalian islets do not scale with the weight of the animal suggests there is an optimal size for the function of these endocrine miniorgans (161, 162). Recapitulating the size of human islets by spontaneous reaggregation (159, 163) or controlled forced aggregation using micropatterned culture plates improves the functionality of stem cell-derived beta cells (160).

The signaling cues required in the later stages of the differentiation protocols to induce maturation are not completely identified. Recent studies have shown that better functioning hPSC-derived beta cells are generated when serum-free media with no added factors is used in the later stages (159, 163). Velazco-Cruz and colleagues reported remarkable acquisition of dynamic glucose stimulated insulin secretion, including robust first and second phases, following the omission of TGF-beta inhibition together with cluster resizing and use of serum-free media during the last stage of the differentiation process. Letting the stem cell-derived islet-like cells establish their own niche and paracrine/autocrine signaling might be a better alternative to achieve more functional cell types (44, 164).

A common problem in the field of hPSC differentiation is the robustness of a given protocol applied to different hPSC lines. In most instances, protocols are optimized specifically for one or few cell lines, and they tend to yield variable differentiation efficiencies when other cell lines are used. In the case of pancreatic differentiation, reports have shown how a particular differentiation protocol results in different percentages of pancreatic progenitors and insulin expressing cell numbers depending on the hPSC line used (148, 165). This is an important obstacle to the wide application of published differentiation protocols, affecting reproducibility. It also complicates the generation of beta cells from diverse patient-derived hiPSCs for disease modeling purposes.

The variability in the efficiency of a particular differentiation protocol has been attributed to the hPSC line genetic background, which can condition its response to inductive cues (166, 167). Recent studies suggest that specific genetic variants in hiPSC lines may alter the differentiation efficiency towards definitive endoderm (168). By using pools of 125 different iPSCs and single-cell RNA sequencing, the authors mapped the population variation during definitive endoderm differentiation stages. They identified several molecular markers predictive of differentiation efficiency, demonstrating that it can be altered by germline genetic variants. Despite intense efforts to improve *in vitro* differentiation protocols, currently they only partially recapitulate the optimal *in vivo* signaling environment. Missing signaling cues are probably better tolerated in some cell lines than in others, explaining this apparent genetic background-determined fitness to respond efficiently to a given protocol. A partial solution to the problem of variability in differentiation efficiency across cell lines is the generation of genome edited cell lines. Genome editing technologies have made possible the introduction and correction of point mutations in hPSCs (169–171). In particular, CRISPR-Cas9 technology has proven particularly useful to efficiently generate isogenic cell line pairs. These can be obtained either by correcting the genetic variant of interest in patient-derived iPSC, or by introducing mutations in a hPSC line that differentiates robustly to the cell type of interest (172, 173). CRISPR can also be used to elucidate which signaling pathways and mechanisms are important to achieve a particular differentiation stage. A recent report illustrates this approach by using a genome-wide CRISPR screening to identify JNK-JUN signaling as a barrier for pluripotency exit and endoderm differentiation (174).

Generation of patient-derived hiPSCs and their differentiations towards the pancreatic lineage has facilitated the generation of cellular models to study diabetes. In combination with genome editing technologies, these approaches make it now feasible to study how a particular genetic variant impacts pancreas development and beta cell physiology. The first diabetes disease modeling studies assessed the ability of patient-derived hiPSCs and healthy donor controls to efficiently differentiate into beta cells (175, 176). CRISPR-Cas9 genome editing has been exploited to correct point mutations associated with diabetes in patient-derived hiPSCs or to generate knockouts (KOs) of critical pancreatic and beta cell genes (177–181). Maxwell and colleagues showed that they were able to generate functional beta cells using a CRISPR-Cas9 edited iPSC line obtained from a patient with *WFS1* mutation. The corrected cells exhibited robust first- and second-phase insulin secretion in response to glucose challenge and restored euglycemia when implanted into diabetic mice, while the unedited controls did not (182).

Diabetes disease modeling studies based on hPSCs have been mostly focused on genes that cause neonatal diabetes, since the expected severe phenotype due to the developmental defect is assumed to be easier to detect. Together with patient-derived hiPSCs, several KO hPSC lines have been genome engineered to study neonatal diabetes disease phenotypes. Several reports have studied the outcomes of disrupting critical pancreatic developmental genes like *NEUROG3* (177), *PDX1* (183), *GLIS3* (184), *RFX6*, *PTF1A*, *MNX1*, *HES1*, *ARX* (178, 185), *GATA4*, *GATA6* (180, 181), or *SIX2* (186). Similar approaches have been exploited to dissect the disease mechanisms behind mutations in *HNF1B* (176) and *HNF4A* (187) that cause MODY or a rare mutation in *STAT3* gene causing neonatal diabetes (179). Genes that harbor genetic variants associated with increased risk of T2D have also been knocked out with CRISPR in hPSCs to study their role in beta cell development and function (e.g. *CDKAL1*, *KCNQ1*, *KCNJ11*) (188, 189).

Beyond genetic defects impairing pancreatic and beta cell development, those directly affecting beta cell insulin secretion are more challenging to study due to the current limitations of the hPSC-based models, in particular the functional immaturity of the derived beta cells. Genetic defects in K^+^_ATP_ channel genes (64, 188, 190), the insulin gene (191, 192), or the ER-stress related genes *WFS1, YIPF5* and *MANF* (79, 80, 175) that cause neonatal diabetes and congenital hyperinsulinism have been modeled with hPSCs using diverse strategies. These include detailed characterization of the *in vitro* obtained pancreatic cell populations, their expression of relevant beta cell markers, their tolerance to different stresses, and their functionality in response to glucose and other secretagogues. Additional characterization with *in vivo* studies allows the assessment of how defective cells respond to systemic environment cues in terms of further differentiation, maturation, and acquisition of regulated insulin secretion. Phenotyping of the implanted cell populations may be particularly useful when the disease mechanisms do not clearly manifest *in vitro*. This is of particular importance when considering the modeling of strict insulin secretion defects, where the functionality of the beta cells generated *in vitro* may be too immature to correctly ascertain a particular phenotype.

In order to model insulin secretion defects reliably, we will need completely functional hPSC-derived beta cells that are as comparable as possible to the ones found in native human islets. Major obstacles in this quest are: i) the lack of differentiation protocols that robustly generate functional beta cells and are widely applicable to any hPSC line; ii) the high variability of human islet preparations, which makes them a problematic gold-standard control to rely upon; iii) the absence of standardized phenotyping methods for hPSC-derived beta cells and human islets which hinders faithful comparison of results across laboratories.

## Characterization of Stem Cell Derived Beta Cells: How Do They Compare to Human Adult Beta Cells?

Beta cell differentiation protocols are technically complex: they have multiple stages, last over a month, and utilize combinations of recombinant proteins and small molecules at different dosages. During the course of any differentiation, many aspects can deviate from the optimal parameters, leading to poor reproducibility and consistency across experiments. In order to minimize experimental variation and minimize costs, laboratories differentiating hPSC usually implement standard operating procedures to prepare culture reagents and execute the differentiation experiments. Current approaches to characterize the outcomes of the hPSC differentiations towards beta cells rely on a battery of methods applied at select stages of the differentiation process. These methods commonly include, but are not limited to, flow cytometry, immunohistochemistry, and RT-qPCR. It is not uncommon for differentiation experiments to fail due to poor definitive endoderm induction, limited expression of pancreatic progenitor markers, or reduced number of INS+ cells. Since the differentiation of one cell type into the next is not 100% efficient, it is critical to address the identity of the cells in the population at given time points. The percentage of cells reaching definitive endoderm stage, the abundance of PDX1+NKX6-1+ pancreatic progenitors, and the fraction of INS+NKX6-1+ cells are examples of common flow cytometry quantifications. They are proxies for the efficiency and quality of the differentiation in terms of achieving bona-fide beta cells. The ultrastructure of hPSC-derived beta cells and human islets has been compared using electron microscopy, using insulin granule morphology as another indicator of beta cell maturity (121, 142, 153, 160).

Current differentiation protocols yield 40–75% INS+ cells in their later stages, although only 20–52% usually represent bona-fide beta cells expressing INS+NKX6-1+ (see an example of differentiation protocol presented in **Figure 2**). Furthermore, the proportions of cell populations can widely vary between experiments and different cell lines (148, 165). One of the important aspects of human pancreatic development that is still poorly understood is the fate allocation of the different endocrine cell types. The timing of NEUROG3 upregulation seems to influence the fate selection of the endocrine precursors (193), which have been shown to be unipotent (194). Endocrine cell fate selection is likely determined by heterogeneous spatiotemporal signals present in the niche of the trunk domain endocrine progenitors. For example, different ligands of the EGF family can modulate the cell-fate selection: betacellulin was reported to promote differentiation into the beta cell lineage when added to mouse embryonic explant cultures (195). This effect was later shown be mediated *via* EGFR-PI3K/AKT-RAC1 signaling resulting in apical polarity inhibition, NOTCH signaling reduction, and induction of NEUROG3 expression (196).

In comparison with the stem cell differentiation outcomes, *in vivo* pancreatic development is also a highly heterogeneous process. Human islet endocrine cell composition varies depending on the islet size and location (161). It is also highly variable across individuals (30) and ages (197). This heterogeneity probably reflects the complexity of endocrine cell fate allocation during development and the plasticity of the pancreas to adapt during the life of the individual to the different metabolic needs.

At the transcriptomic level, gene expression profiling of stem cell-derived islet cells is determined by bulk RT-qPCR or RNAseq at different stages. Sorting of antibody-stained or INS-GFP reporter lines have been used to study beta cell transcriptomes. Several reports have compared the transcriptome of stem cell-derived islet and beta cells with human islets (121, 141, 142, 153, 160, 163, 198). Hrvatin and colleagues compared the transcriptome of stem cell-derived beta cells to both fetal and adult human beta cells (138). RNAseq analyses showed that the INS+ cells generated with that differentiation protocol were transcriptionally closer to fetal beta cells than to adult ones. They had reduced expression levels of genes associated with the functionality and maturation of the beta cell like *PDX1, NKX6-1*, *MAFA*, *GLI3*, and *MNX1.* Recent reports describing the generation of hPSC-derived beta cells with dynamic glucose stimulated insulin secretion (GSIS) have curiously shown that some important mature beta cell markers associated with functionality are expressed at much lower levels compared to adult beta cells (i.e. MAFA, UCN3, SIX3) (159, 163, 186, 198, 199). It is therefore unclear what should be considered a reliable marker of functional maturity for hPSC-derived beta cells, especially when the expression levels of some of these markers are age-dependent, being low in functional juvenile islets and taking years to increase (200).

Arising single cell technologies are generating a new important source of knowledge that can be utilized in the quest of generating better beta cells. Single cell transcriptomics, mass spectrometry, and epigenomics are providing new insights on the development and physiology of pancreas, islets, and beta cells (30, 201–208). Single cell transcriptomics has proven particularly useful to investigate the differentiation of hPSCs into beta cells by providing novel information about the heterogeneity of the stage-specific populations, their differentiation trajectories, the role of putative regulators of fate decisions, as well as a mean to assess the impact of diabetes-associated genetic variants. Using single-cell RT-qPCR, Petersen and colleagues explored the trajectories of stem cell derived pancreatic progenitors differentiating towards beta cells (209). They identified two pancreatic progenitor populations that give rise to “monohormonal” beta cells. This suggests the existence of alternative differentiation routes toward beta cells, *via* a progenitor stage that expresses NKX6-1 before or after NEUROG3 upregulation. Single-cell RNA sequencing approaches enable the transcriptional profiling of thousands of cells simultaneously. Krentz and colleagues used this approach to characterize mouse and hPSC-derived endocrine progenitors (210). Exploiting fluorescent reporter mouse strains and hPSC lines labeling Neurog3 lineages, they described and compared the heterogeneity of the mouse and human endocrine progenitor populations and the gene markers they express. scRNA-seq can also aid in the interrogation of the molecular mechanisms behind mutations causing neonatal diabetes (182, 192). Veres et al. used scRNA-seq to chart the differentiation trajectories of stem cell-derived populations, showing the presence of different endocrine and non-endocrine cell populations (159). Single cell transcriptomic technologies have been exploited to identify surface markers like ITGA1, which can be used to enrich for beta cells (159), or CD9 which can be used as a negative marker of functional beta cells (211). Similarly, single cell RNA sequencing analysis led to the observation that WNT signaling is reduced in endocrine cells compared to pancreatic progenitors. Chemical inhibition of WNT signaling in hPSC-derived progenitors induced differentiation to endocrine cells (212). By performing scRNA-seq on *in vitro* and grafted stem cell derived islets, Augsornworawat and colleagues were able to show that 6-month grafted cells undergo important transcriptomic changes, acquiring a gene expression profile more similar to human adult islets (199). scRNA-seq technologies thus offer a new window into the understanding of how transcriptomic regulation determines cell state. Part of its potential for the development of stem cell differentiation approaches relies on the direct comparison of the *in vitro* cells with their *in vivo* “real” counterpart. Enterprises like the human cell atlas are yielding body-wide datasets of single cell transcriptomic that are being used to benchmark *in vitro* stem cell differentiated cells (213).

The assessment of hPSC-derived beta cell functionality relies on methods established to characterize human islets. The most conventional method is the evaluation of insulin secretion in response to high glucose, either in a static setup or in a dynamic fashion using a perifusion setup. Additionally, different secretagogues can be used to probe the different insulin secretion mechanisms in place: K^+^_ATP_ channel blockers (Tolbutamide), cAMP level modulators (Forskolin, IBMX), GLP1R ligands (Exendin-4, liraglutide), voltage dependent calcium channel agonists (Bay K 8644), non-glucose metabolic substrates (pyruvate, glutamine, leucine), and forced membrane depolarization (arginine, KCl).

The stimulation index (fold increase in insulin secretion from low to high glucose) of hPSC-derived islet cells in static GSIS reported by most studies ranges from 2 to 3, while human islet indexes have a median of about 7 (32). While static glucose stimulated insulin secretion is seemingly a straight-forward assay, there is a wide range of protocols used in the field for both hPSC-derived cells and human islets. They diverge in important critical points: the concentrations of low and high glucose used, the length of the stimulation, the number of cells/aggregates/islets used for the test, the composition of the stimulation buffer, the washing steps, the length of the equilibration period, and the glucose concentration used during that time. Also, the selection of the samples for this assay is not always clearly reported: it is a common practice to hand-pick human islets of homogeneous aspect and size to perform GSIS, which might yield better results than randomly sampled islets. Many of these parameters and details may seem trivial but can introduce important systematic biases that make comparison of results across labs difficult. These comparisons could greatly benefit from the adoption of standardized practices in the functional assessment and phenotyping of both hPSC-derived cells and human islets. Furthermore, a stable positive control could be used in each GSIS test to have a reference point between experiments, but this is usually not possible due to the scarcity and high variability of human islets.

An important characteristic of human islets is their fine-tuned secretion of insulin in response to glucose. This can be clearly observed in dynamic GSIS assays using perifusion systems, where a robust first insulin secretion phase is followed by sustained second phase of insulin secretion with lower output (145). The acquisition of this biphasic insulin secretion pattern does not occur in human islets until birth (214). The levels of basal insulin secretion are also a good indicator of beta cell function. Immature beta cells have reduced glucose threshold for insulin secretion which leads to higher basal insulin secretion levels and a relatively low stimulation index (138, 215). Robust dynamic glucose stimulated insulin secretion of hPSC-derived beta cells has been only recently reported (160, 163). It seems to depend on a combination of abundant insulin positive cells in the aggregates, achieved either by high differentiation efficiencies involving late stage reaggregation in media containing no additional signaling cues and the expression of beta cell maturation marker SIX2 (163, 186), enrichment using fluorescent reporter lines (160) or surface marker antigens (159), followed by forced or spontaneous reaggregation, respectively. Interesting, Hogrebe et al. also showed that dynamic glucose stimulated insulin was achieved in beta cells generated using their planar differentiation protocol (153). A remaining challenge faced with hPSC-derived beta cell GSIS is the lower magnitude of insulin secretion in comparison to human islets. Davis and colleagues demonstrated that the disparity may be due to a metabolic bottleneck in the glycolytic pathway that can be ameliorated when glyceraldehyde 3-phosphate dehydrogenase (GAPDH) and phosphoglycerate kinase (PGK1) activities are bypassed (216).

Additional functional characterization has relied on surrogate indicators of insulin secretion, like the measurements of Ca^2+^ influx into the cytoplasm in response to different stimuli. Calcium imaging can be performed on dispersed individual cells or on whole aggregates/islets. It has been used to assess the function of hPSC-derived beta cells in some studies, showing that although calcium dynamics might be similar in a small fraction of cells, they are not as robust as in primary human islet cells (121, 142, 160). Electrophysiological studies of human beta cells using patch-clamp technique have demonstrated the electrical properties of their membranes in response to different stimuli (105, 217). In a recent study, Camunas-Soler and colleagues exploited Patch-seq technology to generate healthy and diseased human islet single cell transcriptomic profiles linked with their electrophysiological characteristics. This valuable dataset enabled them to generate predictive sets of genes that reliably linked gene expression to beta cell function and identify transcriptional programs that contribute to beta cell dysfunction in type 1 and type 2 diabetes (218). Basford and colleagues examined the electrophysiological properties of beta cells derived from an INS-GFP+ reporter stem cell. Compared to human adult beta cells, stem cell-derived beta cells presented heterogeneous K_ATP_ (45% of the cells) and Ca^2+^ (42% of the cells) channel currents and no Na^+^ channel currents (134). To the best of our knowledge, there are presently no reports of stem cell derived beta cells demonstrating electrophysiological properties identical to those of primary human adult beta cells.

Glycolysis coupled with efficient mitochondrial respiration is required for normal insulin secretion (219). During beta cell maturation, active DNA methylation silences the expression of disallowed genes (e.g. *HK1*, *LDHA*) that interfere with the glucose-secretion coupling (220). Neonatal acquisition of aerobic oxidative metabolism is a crucial step for the maturing beta cell, a process shown to be induced by non-canonical WNT4 signaling and estrogen related receptor gamma (ESRRG) (221, 222). Rates of O_2_ consumption and CO_2_ production can be used to evaluate the respiratory capacity of islets and hPSC-derived cells (223, 224), and serve as both a functionality and maturation surrogate marker in the efforts of making better beta cells (160, 221). Enrichment and reaggregation of hPSC-derived beta cells induced mitochondrial metabolic maturation, and the ultrastructure of mitochondria showed increased folding and stacking of cristae (160).

All hPSC-derived beta cell characterization approaches are ultimately benchmarked against human islets. Unfortunately, human islets typically demonstrate wide phenotypic variability across batches (30, 121). This particular point is frequently not suitably acknowledged, and it is particularly problematic when batches of poorly performing islets are used for the comparison to hPSC-derived beta cell preparations. Systematic evaluation of human islets batches at different levels (cell composition, functionality, transcriptomics, etc.) is a step in the right direction to highlight this variability and define a canonical human islet response (**Table 2**). This is illustrated by the Alberta Diabetes Institute IsletCore database collaborative initiative spearheaded by the MacDonald laboratory (32), where traceable phenotypes of over 300 human islets batches demonstrate the remarkable variation in functionality.

**Table 2:** Key characteristics of human islets.

**Morphology:** spheroid **Number of endocrine cells/islet:** ~1500 **Mean islet size:** ~150 µm **Endocrine architecture and composition:** * Beta cells ~50–60% interspersed throughout the islet * Alpha cells ~40% interspersed throughout the islet * Delta cells ~10% * Gamma and PP cells <1% **Stimulated insulin secretion** * High glucose-stimulated insulin challenge (static GSIS) stimulation index: ~7-fold * High glucose-stimulated insulin challenge (dynamic GSIS): rapid biphasic response * Potentiated secretion in response to cAMP modulators (e.g. Forskolin, IBMX) * Potentiated secretion in response to incretin hormones (e.g. GIP, GLP-1) * Increased secretion in response to membrane depolarization (e.g. KCl) * Increased secretion in response to K_ATP_ channel activators (e.g. tolbutamide) * Increased secretion in response to calcium channel agonists (e.g. Bay K 8644) * Increased secretion in response to non-glucose nutrients (e.g. palmitate, leucine) **Key transcription factors and maturation markers:** SIX2, SIX3, UCN3, MAFA, NKX6.1, INS, PDX1, GLIS3, MNX1 **Morphology of mature insulin granules:** dense core vesicles **Dithizone staining:** brick red color **Respiratory capacity:** primarily mitochondrial **Calcium signaling:** increased pulsatile signaling in response to high glucose

List of various features of primary isolated human islets that can be taken into consideration for comparison to stem cell derived islet cells.

## Modeling Diabetes With Implanted Stem Cell Derived Islet Cells

An alternative approach to derive functionally mature hPSC-derived beta cells is to implant their precursors into immunocompromised host rodents. The first report describing this approach showed that a few months after implantation the grafts secreted human insulin in response to systemic glucose administration (125). Since then, multiple implantation sites (subcutaneous, intramuscular, renal subcapsular space, epididymal fat pad, pancreas) (121, 125, 225, 226) and several animal models (SCID-beige, NSG, NOG, NRG-Akita mice; nude rats) (125, 142, 227) have been employed with variable success.

Implantation in the renal subcapsular space is one of the preferred approaches since it is relatively easy to implant and retrieve the cells months later *via* survival nephrectomy. Upon implantation, cells become vascularized and interestingly their cytoarchitecture can undergo rearrangement (131), concomitantly with an increase in the functional maturation (121, 227). Endogenous pancreatic beta cells in recipient animals can be largely eliminated by administration of a beta cell toxin (e.g. alloxan or streptozotocin) either before or after implantation of hPSC-derived cells. The doses used are typically relatively harmless to the hPSC-derived cells owing to species differences in toxin sensitivity (228, 229). Graft function is monitored by measuring circulating human C-peptide levels (using assays that can distinguish the graft derived human *versus* recipient’s endogenous C-peptide owing to sequence differences), and the response to glucose can be determined with intraperitoneal, intravenous, or oral glucose tolerance tests. Also, hPSC-derived pancreatic progenitors or endocrine cells can be transplanted within macro- or micro-encapsulation devices (132, 230–232).

An interesting phenomenon is the functional maturation of implanted beta cells with time. Rezania and colleagues described the progressive increase in circulating C-peptide levels for several weeks after implanting pancreatic progenitor cells or more matured cells (121, 131), a phenomenon which has also been observed by others (160, 192, 199). Furthermore, Rezania et al. observed faster diabetes recovery and achieved higher circulating C-peptide levels sooner when the implanted cells were further along in their differentiation prior to implant (121). It remains unclear what factors promote the apparently successful maturation of differentiated hPSCs post implant. One possibility is that immature hPSC-derived beta cells require a critical niche and systemic factors including vascularization and proper oxygenation to acquire full functionality (233). Interestingly, maturation of hPSC-derived pancreatic progenitors is accelerated in rats compared with mice (228), something that the authors correlated with increased levels of thyroid hormone in the rats, in line with the fact that thyroid hormone promotes beta cell maturation in rats (143) and in differentiating hPSCs (121). Sex hormones may also influence *in vivo* maturation of pancreatic progenitors since following implant, glucose-stimulated insulin secretion was observed in female mice before males (234). The systemic environment also provides periodic signals, which might entrain the circadian clock of the implanted stem cell-derived islet cells, leading to their functional maturation (235–238).

Several studies reporting the generation of hPSC-based diabetes disease models have relied on implanting mice with hPSC-derived beta cells. This constitutes a practical solution to study the function of the beta cells in a systemic environment, especially when the disease phenotype is not apparent *in vitro* (80, 175, 188, 191, 192). It is also useful to investigate the impairment of development caused by mutations perturbing critical regulators of islet cell development (177, 179, 180). Once grafts have matured, these models offer the possibility to dissect the effect of particular mutations on insulin secretion by carefully examining their responses to different stimuli.

An important aspect after implantation of hPSC-derived beta cells, is their capacity to survive the hypoxic environment of the implantation site until they become vascularized. This is a critical stress period that may result in the apoptosis of the most differentiated endocrine cells (239). Faleo and colleagues reported that this might be partially overcome by acclimatizing the cells to hypoxic conditions before implantation. In this regard, the format of the implanted cells likely also plays a critical role in successful engraftment, with smaller aggregates probably benefiting from faster vascularization kinetics as shown for engineered pseudoislets (240).

Another concern with stem cell derived islet cell implants, especially when using them on diabetic rodents, is the “pellet” effect, in which the non-regulated basal insulin secretion coming from immature beta cells might be enough to rescue hyperglycemia. This brings up the question of how many cells should be implanted to achieve an optimal working graft, which obviously will depend on the stage of differentiation and quality of the cells in terms of functionality (121). The composition and format (e.g. aggregate size) of the implanted cell population is also likely critical for a successful outcome. In fact, the formation of pancreatic progenitor cell aggregates prior to implant was shown to be essential for the formation endocrine cells (132). In order to investigate the *in vivo* maturation process of hPSC-derived beta cells, novel *in vivo* imaging technologies could be exploited. Radio tracer-based imaging of beta cell mass and function could prove to be particularly useful in this regard (241, 242).

Although intra islet paracrine signaling between the different endocrine cell types is crucial for fine-tuned insulin secretion (44, 45, 243), different reports have shown that diabetes can be rescued with nearly pure populations of islet beta cells with different efficiency (244, 245). Nair and colleagues described the implantation of 90% enriched hPSC-derived beta cells using an INS-GFP reporter cell line. While the grafts presented a few polyhormonal (INS+GCG+) cells 3 days after implantation, 8 weeks later, there was clear presence of GCG+ and SST+ monohormonal cells together with the beta cells, suggesting that the polyhormonal cells gave rise to monohormonal alpha and delta cells that likely mediate paracrine signaling contributing to optimal insulin secretion (160). The proportion of endocrine cell types in the implant to achieve the best glycemic control possible is an interesting question that requires further investigation.

Further understanding of the factors playing an important role in the functional maturation of implanted hPSC-derived beta cells will pave the way to the generation of better humanized mouse models to study insulin secretion. Ultimately, optimal control of implantation parameters will reduce the associated variability of these experiments enabling the careful assessment of the impact of genetic variants on insulin secretion.

## Conclusion

**Figure 3:**
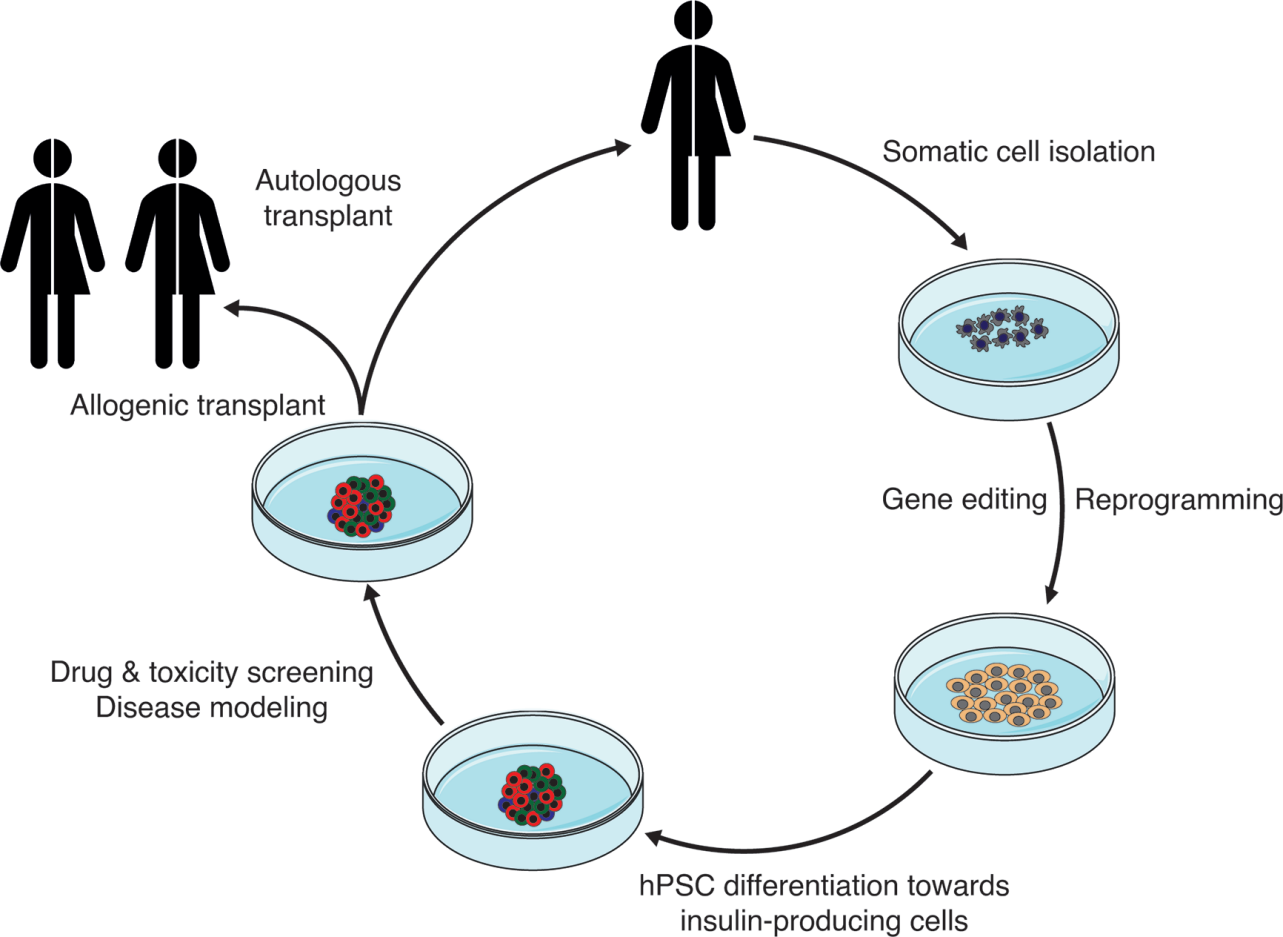
Schematic showing the potential application of stem cell-derived insulin-producing cells for the treatment of diabetes.

Derivation of endocrine islet cells from hPSCs has become an attractive possibility to model diabetes disease and screen for new treatments (**Figure 3**). The progress in the last decade has made it feasible to obtain cells *in vitro* that closely resemble the native adult counterpart. Arising technologies like CRISPR-Cas9 genome editing and single cell transcriptomics are aiding in the generation of more reliable stem cell models, the refinement of the differentiation protocols and the characterization of the resulting differentiated islet cells. There are still important remaining questions in the quest for more functionally mature beta cells: how can we determine and increase beta cell specification? What are reliable mature beta cell markers and the key triggers of functional maturation? Together with detailed single cell transcriptomic characterization, improved characterization of the metabolism, proteomics and functional genomics of the hPSC-derived islets cells, and their comparison with human islets, will certainly pave the way forward. In this common effort, consensus in standardized characterization of the resulting hPSC progeny, the development of robust and reproducible differentiation protocols, and open dissemination of results, will enable prompt replication and speed up the implementation of successful strategies for beta cell generation and diabetes disease modeling.

## Author Contributions

Writing—original draft preparation, DB. Writing—review and editing, DB, DI, and TK. Funding acquisition, TK. All authors contributed to the article and approved the submitted version.

## Conflict of Interest

The authors declare that the research was conducted in the absence of any commercial or financial relationships that could be construed as a potential conflict of interest.
